# Intern Passport: Orienting New Travelers to the Emergency Department

**DOI:** 10.5811/westjem.2018.10.39479

**Published:** 2018-11-13

**Authors:** David A. Masneri, Cedric W. Lefebvre

**Affiliations:** Wake Forest School of Medicine, Department of Emergency Medicine, Winston-Salem, North Carolina

## Abstract

The objective of the Intern Passport (IP) curriculum was to implement a structured orientation for incoming interns that effectively defined and distinguished various personnel and assets within the emergency department (ED). The method of training was an on-the-job orientation that required interns to obtain “stamps” (signatures) on their passports during visits to eight “countries” (specialists) within the ED. Topics covered during the visit included introductions, tasks and capabilities, expectations, and pearls and pitfalls. Interns obtained stamps after spending 30-minute orientation visits with each country during the first four-week rotation of internship. The ED countries visited were Adult Nursing, Pediatric Nursing, Orthopedics Technician, Respiratory Therapy, Pharmacy, Psychiatry, Observation, and Radiology. Effectiveness was assessed by participant completion of an optional anonymous retrospective survey. The IP was a beneficial addition to our intern orientation curriculum. It effectively defined and distinguished various personnel and assets within the ED.

## BACKGROUND

The majority of emergency medicine (EM) residency programs offer formal intern orientation. Didactic sessions and social activities are the most common components of intern orientation, but skills training sessions are increasing in frequency.^1^ Emergency department (ED) access, exposure, and introduction are key components and areas of focus for intern orientation.^1^ During semiannual evaluations, our residency program identified intern self-reported challenges with achieving timely orientation to our ED personnel and resources. Barriers cited included limited number of EM rotations during the first year, large number of specialists and personnel in the ED, and fast pace and restricted free time while working clinically in the ED. A more thorough and structured ED orientation was deemed necessary. We designed the Intern Passport (IP) curriculum to facilitate definition of ED specialists, assets, and resources.

## OBJECTIVE

The primary objective of the IP curriculum was to implement a dynamic, structured orientation program for incoming interns that effectively defined and distinguished various personnel and assets within the ED. Secondary goals were to promote early intern socialization and to develop intern relationships with key personnel. A tertiary goal was to encourage active experiential learning by taking the interns out of the classroom and away from didactic lectures and placing them into the energetic learning and work environment of the ED – the same environment that the interns would call home for the next three years.

## CURRICULAR DESIGN

The IP curriculum was designed to facilitate definition and familiarization of ED specialists, assets, and resources. We structured this innovative method of training as an on-the-job orientation that required interns to obtain “stamps” (signatures) on their passport from eight “countries” (specialists) within the ED. During the intern orientation rotation (the first four-weeks of internship) interns earned a passport stamp after spending a 30-minute orientation visit within each country. The ED countries visited were Adult Nursing, Pediatric Nursing, Radiology, Orthopedics Technician, Respiratory Therapy, Pharmacy, Observation, and Psychiatry. General topics covered during each visit included introductions, tasks and capabilities discussion, specialist expectations, and pearls and pitfalls. We selected this “Feel free to travel about the ED!”-themed design because it actively engaged the mostly-millennial learners, and promoted interactivity via experiential learning in contrast to didactic lectures in the classroom. The IP provided the interns with level-specific training that millennial learners prefer, based on the results published by Shappell and Ahn.^2^

Successful curriculum implementation required motivated participation by both the “travelers” and the “countries.” After we extended an invitation with accompanying explanation of the curriculum design and philosophy, the countries were quick to buy in to this curriculum and eager to get early exposure to the interns. Countries appreciated the opportunity to discuss common errors, order entry (if applicable), pet peeves, and ways to mutually help each other. The style and format of the visits varied based on the country.

Visits were self-scheduled by the interns and were completed as time was available during the orientation four-week rotation. Eight visits of a 30-minute duration totaled four hours of intern time dedicated to this curriculum. Most interns completed visits before or after scheduled orientation clinical ED shifts as a matter of convenience.

All current countries continue to embrace this curriculum and plan continued involvement. Reasons cited included “benefits of early involvement with interns,” “early relationship development,” and “the ability to discuss pearls and pitfalls with ways to help each other.” Additional ED specialists have shown interest, and as a result three additional countries will be added to the travel itinerary for the upcoming intern classes: Research Team, Administration, and Social Work.

## IMPACT

The IP was a beneficial and successful addition to our intern orientation curriculum. A total of 29 out of 30 interns (two consecutive intern classes) completed the IP curriculum. Twenty-four interns completed a retrospective, anonymous, post-participation survey (Survey Monkey®). This study received institutional review board approval at our institution. Of those surveyed, 96% agreed the IP was engaging and relevant to intern orientation; 92% agreed the IP helped establish early relationships and provided a greater understanding and appreciation for ED staff and resources; and 88% agreed the IP made it easier to navigate the ED and locate resources.

Our program plans to continue this easily executed and fun orientation curriculum for future intern orientations. It is beneficial to the” traveler” and “country” alike. The IP may be incorporated into any current EM intern orientation process, and it can be tailored to suit any program, large or small, based on program resources, personnel, and time available.

## References

[1] McGrath J, Barrie M, Way DP (2017). Emergency medicine resident orientation: how training programs get their residents started. West J Emerg Med.

[2] Shappell E, Ahn J (2017). A needs assessment for a longitudinal emergency medicine intern curriculum. West J Emerg Med.

